# Characterization of rectus femoris lesions in knee osteoarthritis at different stages and the effect of ultrasound-guided acupotomy

**DOI:** 10.3389/fphys.2024.1496425

**Published:** 2025-01-23

**Authors:** Wen-Ying Yu, Jing Liu, Ze-Hao Lin, Hong Liu, Liang-Zhi Zhang, Xiang-Long Feng, Zhong-Biao Xiu

**Affiliations:** ^1^ Department of Orthopaedics and Traumatology, The Affiliated People’s Hospital of Fujian University of Traditional Chinese Medicine, Fuzhou, China; ^2^ First Clinical Medical College, Fujian University of Traditional Chinese Medicine, Fuzhou, China; ^3^ Department of Pain, The Affiliated People’s Hospital of Fujian University of Traditional Chinese Medicine, Fuzhou, China; ^4^ Fujian Clinical Research Center for Chronic Musculoskeletal Diseases via Integrative Medicine, Fuzhou, China; ^5^ Key Laboratory of Orthopaedic Injury and Sports Rehabilitation, Fujian University of Traditional Chinese Medicine, Fuzhou, China

**Keywords:** evolutionary pattern, fibrosis, osteoarthritis, rectus femoris, ultrasound-guided acupotomy

## Abstract

**Introduction:**

Chronic injury to the rectus femoris muscle induces and exacerbates the progression of knee osteoarthritis (KOA). However, the lesion characteristics of the rectus femoris muscle in KOA at different stages have not been fully characterized. The aim of this study was to analyze the pattern of lesion characteristics of the rectus femoris muscle at different stages of KOA and to investigate the mechanism by which ultrasound-guided acupotomy operations can prevent and control KOA.

**Methods:**

Early, middle, and late-stage rabbit KOA models were constructed using the modified Videman method. Ultrasonography was used to record the elastic modulus and cross-sectional area of the rectus femoris muscle, and morphology was used to observe the ultramicroscopic changes in the rectus femoris muscle and assess the degree of fibrosis. Additionally, ultrasound-guided acupotomy operations were performed on the rabbit model of late-stage KOA, and alterations in the key molecular markers of rectus femoris fibrosis were determined using Western Blot and qPCR methods.

**Results:**

As the disease progressed, the elastic modulus of the rectus femoris muscle in KOA rabbits gradually increased, the cross-sectional area gradually decreased, and the degree of fibrosis increased. In contrast, the degree of fibrosis in the rectus femoris muscle improved after ultrasound-guided acupotomy intervention.

**Conclusion:**

These findings highlight the gradual increase in the modulus of elasticity, the gradual decrease in cross-sectional area, and the increased fibrosis of the rectus femoris muscle in KOA rabbits as the disease progressed. Ultrasoundguided acupotomy operations have been shown to have a protective effect on KOA cartilage and to delay the progression of KOA by ameliorating pathological changes in the rectus femoris muscle. The mechanism may involve reducing chronic injury to the rectus femoris muscle and protecting joint homeostasis by attenuating the degree of rectus femoris fibrosis.

## 1 Introduction

Knee osteoarthritis (KOA) is a significant health concern among the elderly, representing a comprehensive knee joint disorder that involves cartilage degeneration, subchondral bone sclerosis, synovial inflammation, and changes in the surrounding muscles and ligaments (Hunter and Bierma-Zeinstra, 2019). Early-stage KOA may be asymptomatic or mildly symptomatic and is potentially reversible, whereas end-stage KOA often necessitates total knee replacement surgery (Mahmoudian et al., 2021). The dynamic and complex nature of KOA, along with its biological underpinnings and progression patterns, remain incompletely understood and quantitatively characterized, significantly hindering precise identification and early intervention. Therefore, extracting macroscopic and microscopic indicators and core parameters that characterize KOA, elucidating its patterns of change and biological mechanisms, and exploring effective treatment strategies based on these patterns hold crucial clinical significance (Belluzzi et al., 2019).

KOA is a prevalent chronic musculoskeletal condition affecting over 654 million people worldwide (Duong et al., 2023). Historically, attention has predominantly focused on the degradation of intra-articular cartilage (Hodgkinson et al., 2022). However, changes in the surrounding muscles, notably the atrophy and weakness of the quadriceps, are recognized as critical features of muscle pathology around the knee joint (Li et al., 2024). Chronic low-grade inflammation in KOA suppresses presynaptic reflexes, reducing α-motor neuron activity and leading to quadriceps atrophy and weakness (Mansfield et al., 2022). Additionally, joint pain and mobility impairments in KOA patients can lead to disuse atrophy of the quadriceps (Sherman et al., 2024). This muscle atrophy and weakness, mediated by altered lower limb biomechanics, leads to knee joint overload and accelerates cartilage deterioration, creating a negative vicious circle (Mohajer et al., 2022). Recently, strategies to improve skeletal muscle health have been increasingly recognized as vital for improving the symptoms and functional outcomes of knee osteoarthritis (Smith et al., 2024). Numerous studies have confirmed that enhancing the functional training of the quadriceps can effectively alleviate the progression of KOA (Hansen et al., 2023). The rectus femoris, unique within the quadriceps for crossing both the hip and knee joints, can develop pathologies due to dysfunctional hip-knee coordination and imbalanced force lines in the lower limbs (Murdock et al., 2024). Current research on adjacent knee muscles in osteochondral condition, focuses mainly on their functional role. With limited knowledge of the pathophysiological mechanisms of muscle involvement in KOA progression. Therefore, objectively assessing the characteristics of rectus femoris lesions and identifying typical clinical manifestations are crucial for formulating rehabilitation strategies for KOA.

Under physiological conditions, chronic injuries of the quadriceps mediated by KOA involve degeneration due to inflammatory cell infiltration. During the body’s self-repair process, protective muscle spasms occur, leading to connective tissue proliferation and scarring, which dominate the repair process. This results in skeletal muscle fibrosis, causing disturbances in the circulation of qi and blood and the accumulation of metabolic products, leading to pain (Mukund and Subramaniam, 2020). Therefore, assessing the degree of skeletal muscle fibrosis is crucial for guiding the selection of treatment methods and prognostic judgments for chronic muscle injuries (Mahdy, 2019). Muscle stiffness, or the ability to resist deformation, decreases, which fails to prevent joint laxity, causing joint instability. Previous studies have shown that a decrease in muscle strength impairs muscle function, reflected in reduced muscle stiffness and elastic modulus, as well as disordered collagen alignment. Ultrasound elastography can detect changes in the elastic strain ratio or elastic modulus, reflecting pathological changes in skeletal muscle (Ashir et al., 2023). Previous phases of our research also used elastographic ultrasound to assess the rectus femoris’ elastic strain ratio from the perspective of tissue hardness to evaluate the pathological characteristics of chronic injuries in KOA rabbits. Comprehensive assessments of the evolution of rectus femoris lesions in different stages of KOA are rarely reported.

Ultrasound-guided acupotomy operations utilizes advanced ultrasound technology to perform minimally invasive needle therapy under the guidance and monitoring of ultrasound imaging equipment. Compared to traditional acupotomy therapy, ultrasound-guided acupotomy operations approach offers real-time dynamics, clearly visualizing anatomical structures to avoid blood vessels, nerves, and organ tissues, aiding in diagnosis and precise localization of treatment targets. It significantly reduces treatment risks due to individual differences while validating clinical diagnoses and accurately locating lesions during treatment. Thus, this study aims to establish a rabbit model of KOA, collect multimodal ultrasound images of the rectus femoris’ elasticity and cross-sectional area at different stages, perform quantitative analysis of critical fibrosis indicators of the rectus femoris, and conduct a comprehensive analysis of the characteristics of rectus femoris lesions at various stages of KOA. This will provide experimental evidence for assessing the characteristics of chronic injuries of the rectus femoris in KOA and explore the therapeutic efficacy mechanisms of ultrasound-guided acupotomy operations in treating KOA.

## 2 Materials and methods

### 2.1 Experimental animals and grouping

Fifty-six healthy male New Zealand white rabbits, aged 6 months and with an average body weight of 2.0 ± 0.5 kg, were procured from Shanghai Songlian Experimental Animal Co. Ltd [Production License Number: SCXK (Hu) 2017–0,008]. The Fujian University of Traditional Chinese Medicine Experimental Animal Center [License Number: SYXK (Min) 2019–0,007] facilitated the purchase and subsequent care of these animals. The rabbits were housed individually under natural lighting conditions, with free access to food and water, and a room temperature maintained at 23°C ± 2°C. After a week of acclimatization, 32 rabbits were randomly divided using a random number table method into four groups: control, KOA 2 weeks, KOA 4 weeks, and KOA 6 weeks, with eight rabbits in each group. The remaining 24 experimental rabbits were randomly divided into another “Control, KOA, Acupotomy”, with eight rabbits in each group (Liu et al., 2022). This experiment was approved by the Animal Ethics Committee of Fujian University of Traditional Chinese Medicine (Approval Number: FJTCMIACUC). All procedures throughout the experiment were conducted in strict accordance with the relevant guidelines on the use and ethics of animals.

### 2.2 Preparation and evaluation of the KOA rabbit model

Based on our team’s prior research and modifications to the Videman method (Liu et al., 2020), KOA rabbit models were prepared at different stages, fixed for 2, 4, and 6 weeks, respectively. Behavioral evaluations were conducted using the Lequesne scoring system (Dardenne et al., 2024), and model verification was performed through MRI imaging.

### 2.3 Multimodal ultrasonography of the rectus femoris

Prior to ultrasonography, the experimental rabbits were fasted for 12 h and deprived of water for 4 h. After weighing, atropine was administered subcutaneously at a dosage of 0.1 mg/kg to reduce glandular secretion. The anesthetic mixture was prepared by combining Shu Tai 50 (containing tiletamine and zolazepam) and Suimianxin II (containing ketamine hydrochloride) in a 1:1 volume ratio, administered at a total dose of 0.1 mL/kg (Guo and Li, 2019). Following anesthesia, the left knee joint was scanned using a Hitachi HI VISION Avius L ultrasound system equipped with a high-frequency linear array transducer (5–15 MHz) and Shear Wave Elastography (SWE) mode. Elastic imaging along the long axis and cross-sectional area measurements along the short axis of the rectus femoris were performed. In grayscale mode, the circumference and cross-sectional area of the rectus femoris 2 cm above the patella were measured. The SWE mode was activated, and a square sampling frame was adjusted to avoid blood vessels, placing a circular measurement frame approximately 5 mm in diameter to measure the elastic modulus at 2 cm above the rectus femoris. Measurements were repeated three times to calculate the average value. All ultrasonic images of the rectus femoris were collected by the chief physician of the ultrasound department at our institution, who has over 10 years of experience in musculoskeletal ultrasound examinations.

### 2.4 MRI examination

Following the fixation periods specified by the modified Videman method and the anesthetic above procedure, MRI examinations of the rabbit’s left knee joint were conducted. The rabbits were positioned supine with the knee joint everted, approximately at a 15°angle, with the scanner’s centre focused just below the patella. The scanning sequences and parameters included: T1-tse-cor (FOV: 100 × 100 mm, ST: 2 mm, TR: 831 ms, TE: 11 ms), T2-tse-sag (FOV: 100 × 100mm, ST: 2 mm, TR: 6860 ms, TE: 84 ms), and T2-de-3d (FOV: 130 × 130 mm, ST: 0.6 mm, TR: 19 ms, TE: 9 ms). Changes in the knee joint space, joint effusion, and cartilage surface smoothness before and after modelling were observed. Osteoarthritis grading of the knee joint via MRI was based on the MOAKS scoring system, which correlates the total score with the severity of knee osteoarthritis; A higher score indicates more severe osteoarthritis. Specifically, the scoring included: cartilage defects (0 for none, 1 for <10%, 2 for 10%–75%, 3 for >75%), synovitis (0 for normal, 1 for mild, 2 for moderate, 3 for severe), joint effusion (0 for physiological, 1 for small, 2 for moderate, 3 for large), osteophytes (0 for none, 1 for mild, 2 for moderate, 3 for severe), and other periarticular features such as anserine bursitis, infra-patellar fat pad signal abnormalities, and prepatellar bursa signal abnormalities (1 for present, 0 for absent).

### 2.5 Masson trichrome staining

Under anesthesia, rabbits were euthanized by air embolism at the auricular vein, and the left knee joint was rapidly dissected to obtain the rectus femoris muscle 1–3 cm above the patella, marking the proximal end. The muscle was fixed in 4% paraformaldehyde for 24 h, dehydrated, and a tissue block measuring 0.5 mm³ was cut and embedded in paraffin. Tissue sections, 5 µm thick, were dried in a 60°C oven for 30 min, dewaxed in xylene, and rehydrated through a graded ethanol series. Staining was performed using Weigert’s iron hematoxylin followed by differentiation in hydrochloric acid alcohol and counterstaining with acid fuchsin. After washing in phosphomolybdic acid solution, sections were dehydrated in graded alcohols, cleared in xylene, and mounted with neutral resin. Microscopic examination and photography were conducted, and collagen volume fractions were analyzed and calculated using ImageJ software (collagen volume fraction = area of collagen in the field of view/total field of view area).

### 2.6 HE staining

Cartilage tissues were fixed in 4% paraformaldehyde for 48 h and decalcified with 10% EDTA before routine paraffin embedding. Muscle tissues were similarly fixed and embedded. Both tissue types were sectioned at a thickness of 5 µm. Following deparaffinization in xylene and rehydration through a graded ethanol series, staining was performed with hematoxylin, followed by differentiation with hydrochloric acid ethanol, bluing in phosphate-buffered saline (PBS), counterstaining with eosin, dehydration in graded ethanol, clearing in xylene, and mounting with neutral resin. Completed HE slides were examined under a light microscope.

### 2.7 Ultrasound-guided acupotomy operation

**FIGURE 1:**
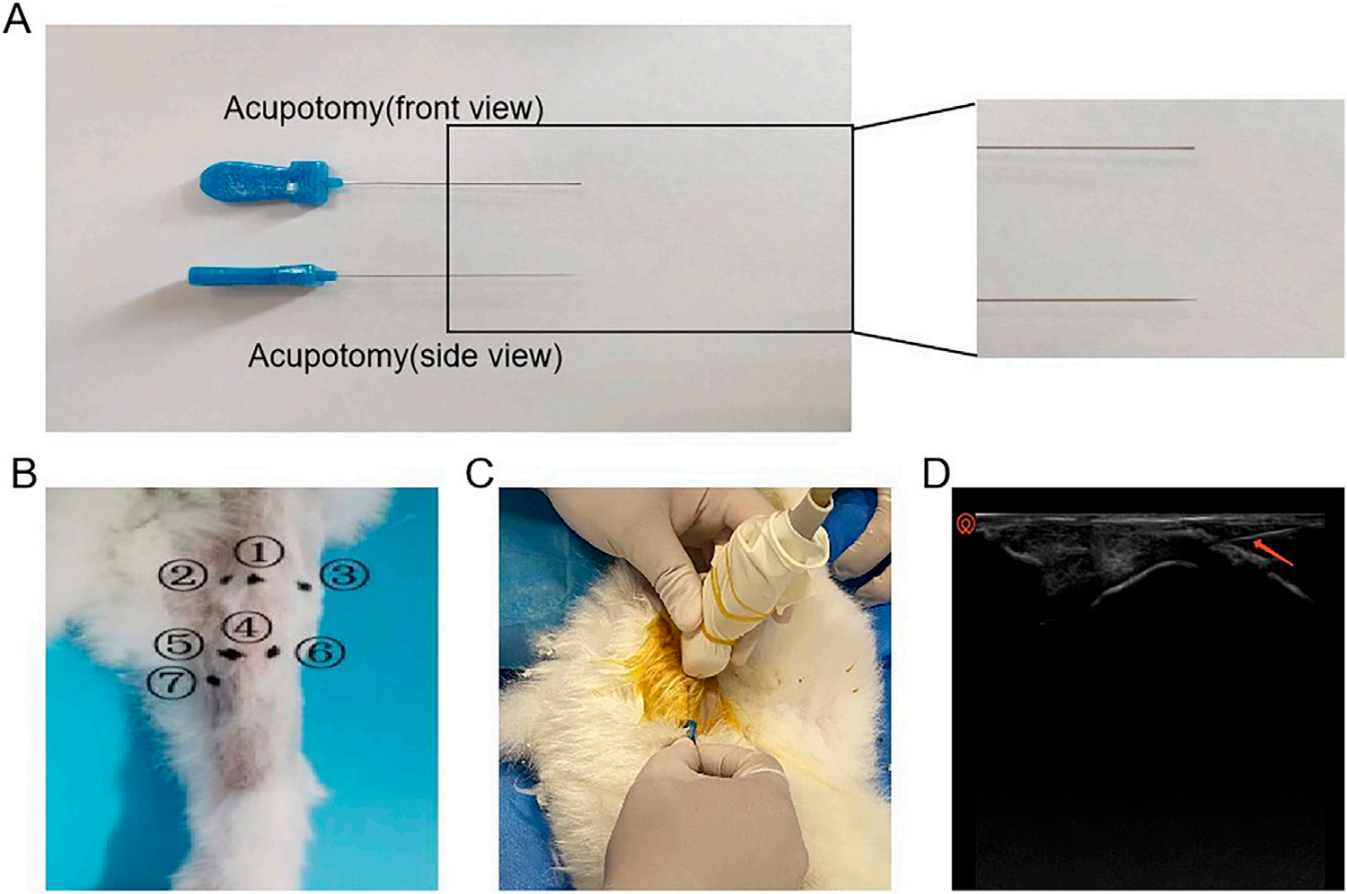
Schematic diagram of the operation of acupotomy intervention. **(A)** Schematic diagram of the acupotomy. **(B)** Intervention point ① He Din Ci; ② Bin Nei; ③ Bin Wai; ④ Bin Xia; ⑤ Bin Nei Xia; ⑥ Bin Wai Xia; ⑦ Yin Ling Ci. **(C)** Visible acupotomy operations. **(D)** Ultrasonographic picture of the acupotomy operation (The red arrow in the picture shows the acupotomy).

After preparing the surgical site, the rabbit was secured on the operating table. Using the nomenclature and localization methods for knee joint myofascial lesion points (see the Supplementary Table S3) described in *Zhong Guo Jing Jin Xue* (Xue, 2012). and referencing the anatomical measurements of the rabbit’s skeletal structure, the Hitachi HI VISION Avius L ultrasound device (high-frequency probe, 5–15 MHz) was employed along with palpation to precisely locate the myofascial lesion points and determine the needle insertion sites. During the procedure, the long axis of the ultrasound probe was aligned with the direction of the muscle fibers, and an in-plane approach was utilized. The blade of the acupotomy was positioned parallel to the direction of the muscle fibers. Under ultrasound guidance, the acupotomy was precisely advanced to the target myofascial node, where small-amplitude lifting, thrusting, and cutting motions were performed. The ultrasound-guided acupotomy intervention is shown in Figure 1. Throughout the procedure, the position of the acupotomy was continuously adjusted under real-time ultrasound guidance to avoid damage to nerves and blood vessels. At the end of the procedure, the acupotomy was immediately withdrawn, and the insertion site was compressed to achieve hemostasis. The treatment was administered once per week for a total of 4 weeks.

### 2.8 Statistical methods

All tests were conducted in triplicate at a minimum. Continuous variables are expressed as mean ± standard deviation (SD). Within-group comparisons were performed using one-way analysis of variance (ANOVA), while pairwise comparisons between groups were conducted using the LSD-t test. The relationships between fibrosis indicators, MOAKS scores, and the elastic modulus and cross-sectional area of the rectus femoris were analyzed using Spearman’s correlation analysis. A *p*-value of less than 0.05 was considered statistically significant. All statistical analyses were performed using GraphPad Prism-8 software.

## 3 Results

### 3.1 The modified videman method successfully induced rabbit models of KOA at different stages

**FIGURE 2:**
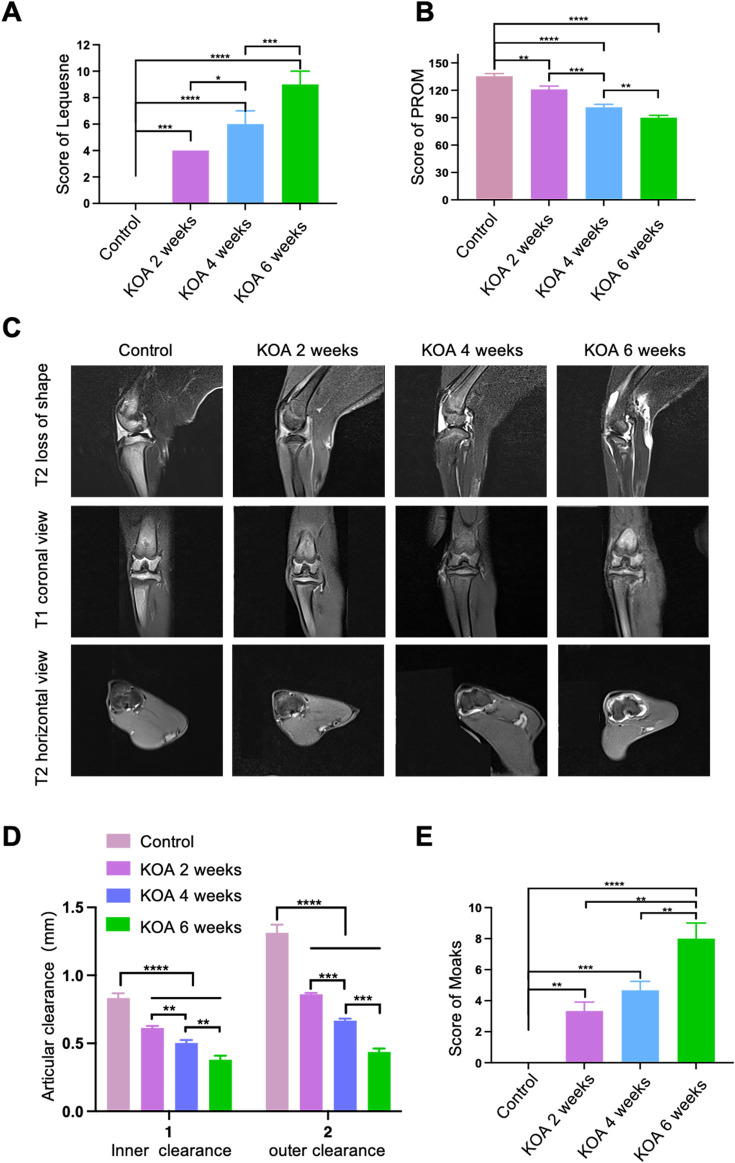
Successful Induction of KOA Rabbit Models at Different Stages Using the Modified Videman Method. **(A)** Scores from the Lequesne MG assessment of the left knee joint in rabbits. **(B)** Passive Range of Motion (PROM) scores for the left knee joint. **(C)** MRI images of the rabbit knee joints. **(D)** Measurements of the medial and lateral joint space in each group. **(E)** MOAKS scores for each group. The control group is labelled as “Control”, representing the baseline comparison. “KOA 2 weeks” represents rabbits modelled for 2 weeks using the Videman method, “KOA 4 weeks” for 4 weeks, and “KOA 6 weeks” for 6 weeks of modelling. Statistical significance is indicated by *p < 0.05, **p < 0.01, ***p < 0.001, ****p < 0.0001, highlighting significant differences between the modelled groups and the control over time.

MRI imaging results indicated that as the modelling period extended, the progression of KOA in rabbits worsened. Before conducting MRI assessments, the animals were evaluated with the Lequesne MG scoring for the left knee joint and knee joint passive range of motion (PROM) assessments. As shown in Figure 2A, the Lequesne MG scores for the left knee joint of all groups were 0 before modeling; after 2, 4, and 6 weeks of modeling, the Lequesne MG scores for the model groups were all greater than 4 points. With the increase in modeling time, the Lequesne MG score continued to rise, and the differences between groups were statistically significant. Moreover, as the modeling time increased, the PROM of the left knee joint gradually decreased (Figure 2B). These results reflect the continuous progression of KOA as the duration of the modelling fixation increased. Further MRI examinations confirmed that with the extension of the modelling time, there was an increase in femoral condyle cartilage defects, more joint effusion, and narrowing of the joint space in the modelled groups, with a significant rise in MOAKS scores (Figures 2C–E). Together, these results demonstrate that we successfully replicated rabbit models of KOA at different stages, and the severity of KOA continuously intensified with the extension of the modelling time.

### 3.2 Changes in rectus femoris stiffness and cross-sectional area across different stages of KOA in rabbits

**FIGURE 3:**
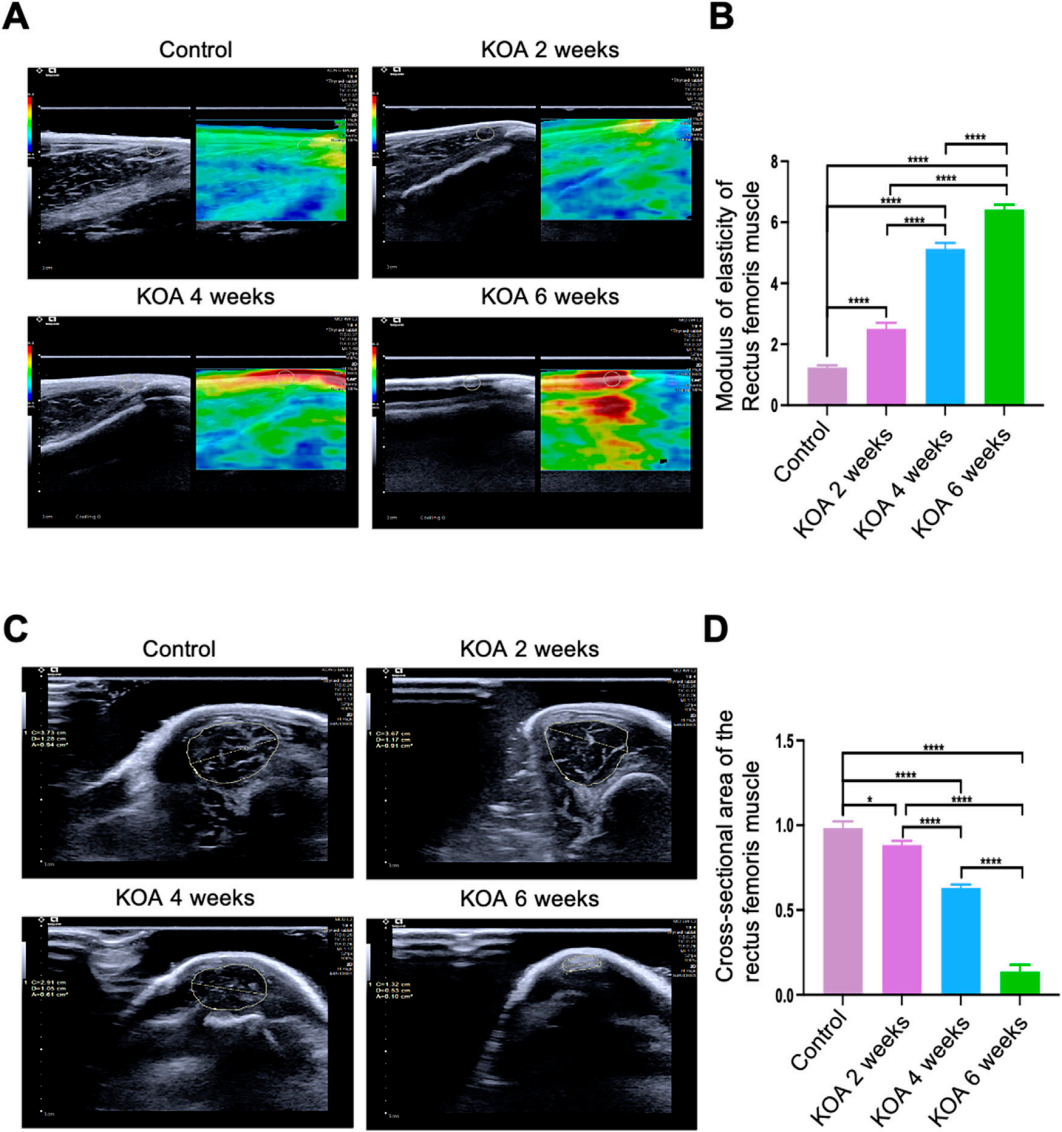
Changes in Rectus Femoris Stiffness and Cross-Sectional Area at Different Stages of KOA in Rabbits. **(A)** Ultrasound elastography images of the rectus femoris 2 cm above the patella in the left knee joint. **(B)** Bar graph showing the elastic modulus of the rectus femoris at the same location. **(C)** Ultrasound images showing the cross-sectional area of the rectus femoris 2 cm above the patella in the left knee joint. **(D)** Bar graph illustrating the cross-sectional area of the rectus femoris at the exact location. Statistical significance is denoted by *p < 0.05, ****p < 0.0001.

The modified Videman method induced significant pathological changes in the ultrasonographic morphology of the rectus femoris muscle in the knee joint. As illustrated in Figure 3A, the control group’s rectus femoris exhibited a predominantly green high-elasticity image. In contrast, the group of KOA 2 weeks showed a high-to-medium elasticity image with primarily yellow and some red hues. The group of KOA 4 weeks group displayed a medium elasticity image with mostly red and some yellow, and the KOA 6 weeks group presented a predominantly red low-elasticity image. Further quantitative analysis of the elastic modulus values among the groups showed that the stiffness of the rectus femoris gradually increased as the modelling period extended, with the elastic modulus values rising progressively,compared to the 2-week modeling group, the stiffness in the other two groups was significantly increased. Furthermore, compared to the 4-week modeling group, the stiffness in the 6-week group showed a further increase, with statistical significance observed between the two. (Figure 3B). As shown in Figures 3A, C, D a significant negative correlation was observed between the cross-sectional area of the rectus femoris and the duration of modelling across the KOA groups. The cross-sectional region of all model groups were significantly smaller than those in the control group, with statistically significant differences (*p* < 0.05 or *p* < 0.0001), indicating a progressive reduction in muscle size correlating with the advancement of KOA.

### 3.3 Histopathological changes in the rectus femoris muscle at different stages of KOA

**FIGURE 4:**
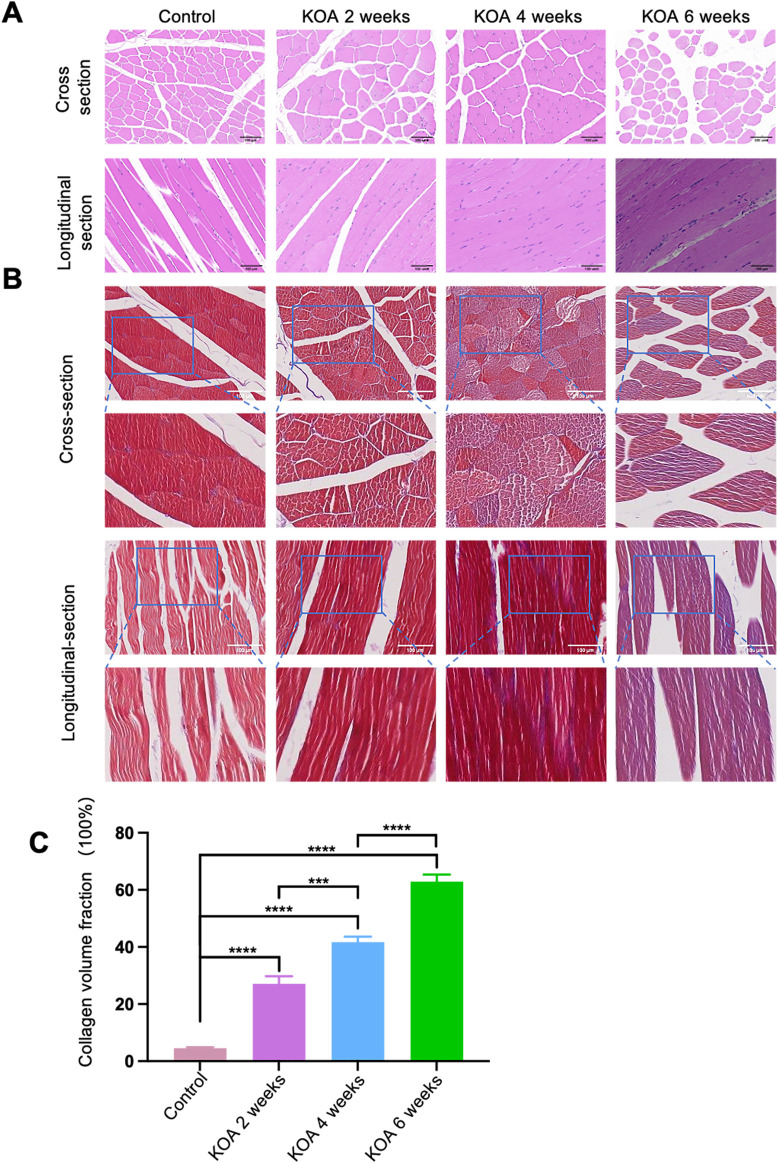
Histopathological Changes in the Rectus Femoris Muscle at Different Stages of KOA in Rabbits. **(A)** HE staining images show both cross and longitudinal sections of the rectus femoris muscle 2 cm above the patella in the left knee joint. **(B)** Masson trichrome staining images of the same sections, highlighting collagen distribution. **(C)** Bar graph derived from Image J software analysis calculating collagen volume fractions (collagen volume fraction = area of collagen in the field of view/total field of view area). Statistical significance is denoted by *p < 0.05, **p < 0.01, ***p < 0.001, ****p < 0.0001.

KOA induced significant pathological changes in the knee joint at different stages. As shown in Figures 4A, B, histological staining of the rectus femoris muscle was performed using both HE and Masson trichrome techniques. The staining results revealed that in the control group, the skeletal muscle fibrosis were well-aligned and stained red. In the KOA 2 weeks model group, the fibrosis were relatively well-aligned with scattered dot-like infiltrations of inflammatory cells and sporadic blue-stained collagen fibrosis. By the 4-week stage, the arrangement of the muscle fibrosis became disordered, with increased infiltration of inflammatory cells and surrounded by a small amount of blue-stained collagen fibrosis. In the 6-week model group, the disarray of muscle fibrosis was more pronounced, with extensive infiltration of inflammatory cells and a large amount of blue-stained collagen fibrosis enveloping them.

Further analysis using ImageJ software on the Masson-stained sections quantified the collagen volume fractions. The results (Figure 4C) showed that the collagen volume fractions in the 2, 4, and 6 weeks model groups were significantly higher than the control group (*p* < 0.001), with the KOA 6 weeks group exhibiting the highest increase, followed by the 4-week and then the 2-week groups (*p* < 0.0001 for KOA 6 weeks vs. others; *p* < 0.001 for KOA 4 weeks vs. KOA 2 weeks). These results indicate that KOA induces pathological changes in rectus femoris tissue, and these changes progressively worsen over time.

### 3.4 Progression of fibrosis in the rectus femoris muscle in KOA rabbits over time

**FIGURE 5:**
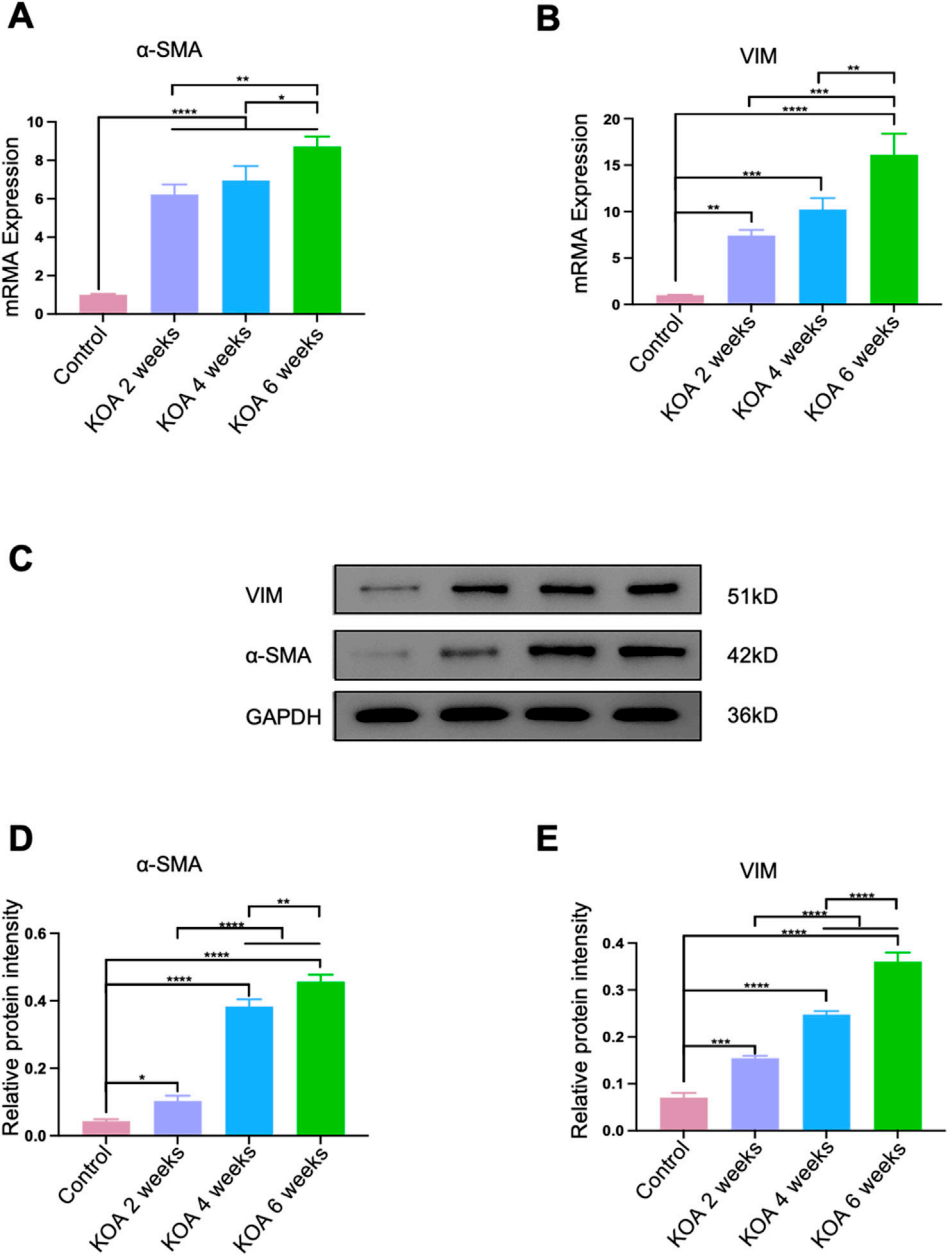
Progression of Fibrosis in the Rectus Femoris Muscle Induced by KOA at Different Stages in Rabbits. **(A)** Bar graph showing qPCR analysis of α-SMA mRNA levels in the rectus femoris. **(B)** Bar graph showing qPCR analysis of VIM mRNA levels in the rectus femoris. **(C)** Western blot electrophoresis images showing the protein levels of α-SMA and VIM. **(D)** Bar graph displaying Western blot analysis of α-SMA protein expression. **(E)** Bar graph displaying Western blot analysis of VIM protein expression. Statistical significance is indicated by *p < 0.05, **p < 0.01, ***p < 0.001, ****p < 0.0001.

The fibrosis key molecular biology markers in the rectus femoris muscle 2 cm above the patella were analyzed using qPCR and Western blotting techniques. As shown in Figures 5A, B, D, E, compared to the control group, the fibrosis markers α-SMA and VIM continuously increased in the KOA groups. From 2 to 4 weeks post-modeling, the progression of fibrosis increased gradually and at a relatively slow pace. In contrast, from 4 to 6 weeks, the degree of fibrosis escalated significantly, showing continuous aggravation with strong statistical significance (*p < 0.05, **p < 0.001, ***p < 0.001, ****p < 0.0001). This could be attributed to the early stage of KOA, where the mildly injured rectus femoris retains a certain level of repair capacity, partially counteracting fibrosis development. However, as the disease progresses to the late stage, prolonged inflammation and stress-induced damage diminish the muscle’s repair capacity, leading to cumulative and pronounced fibrosis progression.

### 3.5 Correlation between the severity of fibrosis in the rectus femoris muscle and the severity of KOA

Skeletal muscle fibrosis is a hallmark of muscle atrophy, aging, and severe muscle damage. To verify the correlation between fibrosis of the rectus femoris and KOA, an initial analysis was performed using MOAKS scores, which assess the severity of KOA, and the fibrosis markers α-SMA and VIM mRNA. As shown in Table 1, α-SMA and VIM mRNA expressions showed a significant positive correlation with the MOAKS scores (r = 0.9381 and r = 0.9664, *p* < 0.0001). This indicates that more significant fibrosis in the rectus femoris is associated with more severe KOA. Considering the difficulty in collecting clinical sample tissues, further correlations were analyzed between the cross-sectional area and the elastic modulus of the rectus femoris with the fibrosis markers α-SMA and VIM mRNA. Meanwhile, Table 1 shows a significant negative correlation between α-SMA, VIM mRNA expressions, and the cross-sectional area of the rectus femoris. As the cross-sectional area decreases, the expression of fibrosis markers increases (r = −0.9091, *p* = 0.0001; r = −0.9021, *p* = 0.0002). There is also a significant positive correlation between α-SMA, VIM mRNA expressions, and the elastic modulus of the rectus femoris; as the expression of these markers increases, the elastic modulus values and tissue stiffness also increase (r = 0.9510, r = 0.9455, *p* < 0.0001).

**TABLE 1:** Spearman correlation analysis between fibrosis markers, MOAKS scores, and rectus femoris elastic modulus and cross-sectional area.

Fibrosis indicators	Score of moaks	Cross-sectional area of the rectus femoris muscle	Modulus of elasticity of the rectus femoris muscle
	Spearman r *p*-value	Spearman r *p*-value	Spearman r *p*-value
α-SMA	0.9381 < 0.0001	−0.9091 = 0.0001	0.9510 < 0.0001
VIM	0.9664 < 0.0001	−0.9021 = 0.0002	0.9455 < 0.0001

These correlation analyses reveal that the severity of KOA is positively correlated with the degree of fibrosis and the elastic modulus of the rectus femoris, and negatively correlated with its cross-sectional area. This relationship can be assessed without the need for clinical tissue collection by using multimodal ultrasound data (elastic modulus and cross-sectional area of the rectus femoris), which reflects the degree of fibrosis in the muscle. These results provide experimental evidence for assessing the pathological characteristics of chronic injury in the rectus femoris associated with KOA.

### 3.6 Ultrasound-guided acupotomy operations mitigates rectus femoris fibrosis and slows cartilage degeneration in osteoarthritis

Acupotomy, a minimally invasive Traditional Chinese Medicine technique, integrates the dual effects of acupuncture and incision. It combines traditional Chinese acupuncture with modern surgical concepts to relax adhered soft tissues through insertion and cutting, without damaging normal anatomical structures or tissue functions. This therapy aims to adjust the body’s biomechanical balance, thereby preventing and treating diseases. Ultrasound-guided acupotomy therapy utilizes advanced ultrasound technology to guide and monitor the minimally invasive procedure.

**FIGURE 6:**
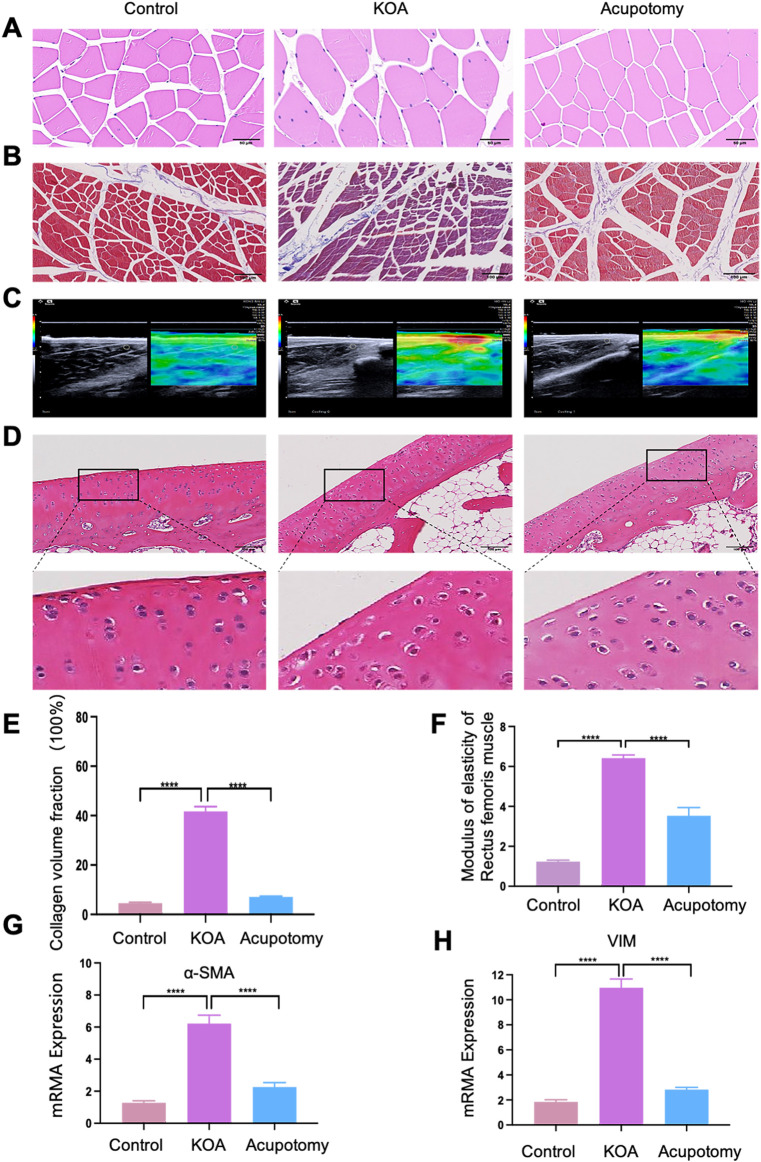
Ultrasound-guided Acupotomy Reduces Fibrosis in the Rectus Femoris and Slows Cartilage Degeneration in Osteoarthritis. **(A)** Representative HE staining image of the rectus femoris. **(B)** Representative Masson trichrome staining image of the rectus femoris, highlighting collagen fibrosis. **(C)** A representative ultrasound elastography image of the rectus femoris shows tissue elasticity. **(D)** Representative HE staining image of cartilage, detailing the structural integrity and cellular arrangement. **(E)** Bar graph of the collagen volume fraction from Masson staining, quantifying the extent of collagen deposition. **(F)** Bar graph showing the elastic modulus of the rectus femoris 2 cm above the patella, indicating tissue stiffness. **(G)** Bar graph of qPCR analysis for α-SMA mRNA expression, a marker of fibrosis. **(H)** Bar graph of qPCR analysis for VIM mRNA expression, another fibrosis marker. Statistical significance is indicated by *p < 0.05, **p < 0.01, ***p < 0.001, ****p < 0.0001.

As shown in Figure 6, compared to the control group, the KOA group exhibited a disordered arrangement of rectus femoris muscle fibrosis, extensive infiltration of inflammatory cells (Figure 6A), and was enveloped by a significant amount of blue-stained collagen fibrosis (Figure 6B). There was an increase in the collagen fraction (Figure 6E) and the elastic modulus (Figures 6C, F), and upregulated expression of fibrosis markers α-SMA and VIM mRNA (Figures 6G, H), indicating an intensified degree of fibrosis. After acupotomy treatment, the arrangement of muscle fibrosis in the rectus femoris was more orderly and closer to normal; the infiltration of inflammatory cells was reduced. Surrounded only by a small amount of blue-stained collagen fibrosis, the collagen fraction was lower than in the KOA group, as was the elastic modulus of the rectus femoris, indicating reduced tissue stiffness. Furthermore, the expression of fibrosis markers α-SMA and VIM mRNA decreased compared to the KOA model group, suggesting a reduction in the degree of fibrosis.

Cartilage HE staining results (Figure 6D) show that the cartilage surface structure of the control group was intact, with a thick cartilage layer, clearly visible cell nuclei, and numerous deep-layer chondrocytes arranged orderly. In contrast, the KOA model group displayed a thinned and rough cartilage layer, reduced number of cartilage cells, disorganized cell arrangement, and unclear layering, with most cells swollen and nuclei wrinkled, ruptured, or vanished. In the acupotomy group, the cartilage surface was smoother, nuclei were more precise, the number of deep-layer chondrocytes was higher, and the arrangement was orderly with less nuclear wrinkling. These results indicate that KOA causes pathological changes and increased fibrosis in the rectus femoris. Acupotomy can partially reverse these pathological morphological changes, alleviate fibrosis, and thus delay cartilage degeneration.

## 4 Discussion

Before this study, there had been no investigations into identifying the chronic injury characteristics of the rectus femoris in KOA from the perspective of multimodal ultrasound imaging combined with fibrosis. Here, we report that the rectus femoris in KOA presents distinct pathological features over time. Using the modified Videman method to replicate KOA models, we observed that as the fixation time increased, the cross-sectional area of the quadriceps progressively decreased, the elastic modulus increased, and the hardness and degree of fibrosis intensified. These findings are highly consistent with the severity of KOA as determined by MRI examinations. Furthermore, our results show that applying ultrasound-guided acupotomy to loosen fibrotic lesions can reduce the degree of rectus femoris fibrosis and slow cartilage degeneration, thereby providing experimental evidence for assessing the chronic injury characteristics of the rectus femoris in KOA and substantiating the importance of rectus femoris pathology in the progression of KOA disease. This also provides a rehabilitation treatment strategy for using ultrasound-guided acupotomy to alleviate rectus femoris fibrosis and delay the progression of KOA.

Previous evidence suggests that as a weight-bearing joint, the knee largely relies on mechanical signals to maintain joint homeostasis, and abnormal mechanical loads play a crucial role in initiating and progressing KOA (Segarra-Queralt et al., 2024). Mechanical loads from physiological movement are vital for maintaining knee joint health, while abnormal loads can lead to dysfunctional cartilage cells and metabolic imbalances, triggering osteoarthritis. Abnormal mechanical performance of the per knee skeletal muscles is considered a primary factor in the onset of KOA (Lopes et al., 2024). In many clinical guidelines for osteoarthritis, exercise therapy is recommended as a core part of managing KOA pain (Nakanishi et al., 2024). However, the mechanisms of skeletal muscle in the pathophysiology of KOA are not yet precise, so understanding the pathologic features and mechanisms of KOA skeletal muscle can aid in formulating clinical rehabilitation strategies and predicting the effectiveness of interventions to prevent further disease progression.

KOA is a common musculoskeletal disorder characterized primarily by knee joint pain, swelling, and restricted movement. KOA encompasses oral non-steroidal anti-inflammatory drugs (NSAIDs), intra-articular injection therapies, and surgical interventions (Sattari et al., 2024; Siviero et al., 2020). Among these, Total knee arthroplasty (TKA) and Unicompartmental knee arthroplasty (UKA) are the predominant surgical options for patients with end-stage KOA. Evidence indicates that knee arthroplasty significantly improves physical function and reduces pain, with TKA demonstrating superior efficacy in patients with advanced KOA (Hu et al., 2024). However, growing research underscores the importance of comprehensive preoperative assessments in optimizing postoperative recovery, while tailored rehabilitation strategies following surgery are pivotal in achieving optimal therapeutic outcomes (Zhang et al., 2024). The quadriceps, as the most important muscle group for extending the knee, is considered a critical risk factor for the degeneration of KOA cartilage, compared to other muscle groups (Chang et al., 2022). There is compelling evidence, including recent clinical guidelines (Xiu et al., 2023) and a systematic review (Hansen et al., 2023), that exercising the quadriceps can alleviate the pain and disability caused by osteoarthritis. Tissue damage disrupts the mechanical homeostasis that is the basis of typical tissue structure and function. If damage is unresolved and homeostasis is not restored, it can lead to a continuous change in the mechanical environment due to pathological matrix deposition and hardening, resulting in progressive fibrosis (Taherian et al., 2024). Therefore, assessing the degree of quadriceps fibrosis is crucial for guiding the selection of treatment methods and prognostic judgments for chronic quadriceps injury in KOA. Ultrasound provides real-time multi-planar imaging without the need for exposure to ionizing radiation or contrast agent administration, and it can sensitively and specifically identify soft tissue and superficial bone changes, offering advantages for non-invasive vascular assessment. It has already begun to be used in the adjunctive diagnosis of osteoarthritis (Johnson et al., 2024). Previous studies have confirmed that elasticity imaging can reflect pathological changes in the quadriceps by measuring the elastic strain ratio or modulus values (Ciuffreda et al., 2024). However, before this study, the evolving patterns of pathological features in KOA rectus femoris had not been revealed, and we have now provided supportive evidence that the chronic injury severity of the rectus femoris in KOA displays distinct pathological patterns over time. As the disease progresses, the cross-sectional area of the rectus femoris decreases, the elastic modulus increases, and the degree of fibrosis gradually intensifies.

Long before this study, research had confirmed that the modified Videman method, involving plaster fixation of the left hind limb in an extended position, passively shortens the quadriceps due to sustained knee extension, leading to circulatory disruptions and muscle atrophy (LongFei et al., 2023). Concurrently, the knee cartilage faces persistent abnormal stress, resulting in abnormal cartilage metabolism and exacerbated cartilage degeneration, thereby simulating the chronic injury of periarticular soft tissues and the degeneration process typical in KOA, unaffected by surgical trauma or inflammation. This method is a commonly used technique for creating ideal KOA models (Gan et al., 2023). The modified Videman method has been selected as a model for KOA primarily due to its ability to accurately simulate mechanical injury or abnormal loading, thereby replicating pathogenic mechanisms observed in human KOA, such as excessive mechanical stress and cartilage degeneration. This feature enhances its relevance to clinical research and facilitates in-depth investigations into underlying mechanisms. Furthermore, the modified method streamlines experimental procedures, minimizes errors, and improves the reproducibility and reliability of experimental outcomes. Lastly, this approach causes relatively minor harm to animals, adheres to ethical standards for animal experimentation, and is well-suited for widespread application in KOA research. Studies have shown that using the Videman method to fix the rabbit’s left hind limb in an extended position for 2, 4, and 6 weeks can mimic the early, middle, and late-middle pathological stages of KOA (LongFei et al., 2023). In selecting the stages of KOA, we compared the pathological features observed in experimental animals with the clinical classification of human KOA (Zeng et al., 2023). This model focuses on the early and intermediate stages of cartilage degeneration, as these stages represent critical intervention windows for KOA in clinical practice. While the model does not fully replicate the entire progression of human KOA, it effectively captures the key pathological changes characteristic of these specific stages. Therefore, this study continued with the team’s previous improvements on the Videman method to prepare rabbit models of KOA at different stages, aiming to assess the characteristic pathologies of the rectus femoris at various stages. The successful creation of models at different stages of osteoarthritis was also confirmed through Lequesne MG scoring, PROM testing, and MRI examinations.

KOA exerts a wide range of clinical effects on the human body. The resulting joint dysfunction severely limits patients’ ability to perform fundamental daily activities, such as walking and ascending or descending stairs, leading to a profound deterioration in quality of life. Persistent joint pain and inflammation not only complicate pain management but also contribute to the development of mental health disorders, including depression and anxiety (Yang et al., 2024). Additionally, KOA is strongly associated with systemic complications, such as weight gain, an increased risk of cardiovascular diseases, and muscle atrophy, which collectively intensify the overall health burden (Mohajer et al., 2022; Shumnalieva et al., 2023). The prevalence and severity of KOA are notably higher in women, particularly after menopause, when declining estrogen levels accelerate cartilage degeneration. Furthermore, aging intensifies the progressive deterioration of both cartilage and bone density (Biz et al., 2024). Therefore, a comprehensive treatment plan for KOA should prioritize a clinical rehabilitation strategy that seamlessly integrates holistic care with tailored, patient-specific approaches. In assessing the pathological changes in the rectus femoris at different stages of KOA, we observed changes in various aspects such as pathological injury, muscle cross-sectional area, and elasticity as KOA progressed. Specifically, the rectus femoris exhibited reduced cross-sectional area, increased elastic modulus, intensified fibrosis, and exacerbated pathological injury. The degree of quadriceps atrophy is closely related to the severity of KOA (Hansen et al., 2023; Wu et al., 2024) and our findings indicate a gradual decrease in the rectus femoris cross-sectional area as KOA progresses, serving as solid evidence of muscle weakness and atrophy consistent with other scholars’ previous findings (Ye et al., 2024). Beyond highlighting the crucial role of rectus femoris atrophy and weakness in KOA progression, some researchers also consider excessive stiffness of the quadriceps as a significant risk factor for knee joint disorders (Ebihara et al., 2020). Clinical experiments have shown that the stiffness of the lateral quadriceps in KOA patients is higher than in healthy individuals, and they found a significant positive correlation between lateral quadriceps stiffness index and WOMAC scores (Xiu et al., 2023), aligning with our findings of increased rectus femoris elastic modulus and stiffness as KOA progresses. Specifically, the control group and the KOA 2 weeks model group showed predominantly green elasticity images with some yellow, the 4 weeks model group displayed red and yellow intermixed elasticity images, and the 6 weeks model group showed predominantly red elasticity images. Clearly, the stiffness of the rectus femoris is positively correlated with the progression of KOA. Furthermore, we observed an increasing degree of fibrosis in the rectus femoris as KOA progressed. Skeletal muscle fibrosis impairs muscle function, negatively affects muscle regeneration after injury, and increases susceptibility to further injury, thus being considered a primary cause of muscle weakness. Previous studies found that compared to the control group, the extracellular matrix (ECM) was significantly enlarged in moderate KOA patients, which they believed limited muscle regenerative capacity (Long et al., 2023). Further experiments revealed a negative correlation between quadriceps strength and fibrosis collagen deposition (Noehren et al., 2018). Skeletal muscle fibrosis is a marker of muscle atrophy, aging, and severe muscle injury. A view also confirmed by Kim (Kim et al., 2023). Recent animal models have shown that excessive fibrosis can impair overall muscle strength. These results corroborate the close relationship between fibrosis and skeletal muscle strength, which is consistent with our findings. Our results indicate that as KOA progresses, the collagen volume fraction of the rectus femoris gradually increases. With extended modelling time, disarray in muscle alignment intensifies, inflammatory cell infiltration worsens, and fibrosis deepens. The degree of pathological injury in the rectus femoris is closely linked to the disease staging of KOA. Our findings reveal the chronic injury pathology evolution pattern in KOA skeletal muscles, showing that as the disease progresses from early to late stages, the elastic modulus of the rectus femoris gradually increases, its cross-sectional area decreases, and the degree of fibrosis intensifies.

Ultrasound-guided acupotomy therapy enhances this approach by integrating dynamic ultrasound guidance, making the procedure visible, precise, and safe. This technique provides dynamic visualization support for preoperative diagnosis, intraoperative guidance, and *p* ostoperative assessment of acupuncture therapy. Compared to traditional methods, visual acupotomy utilizes ultrasound to probe pathological tissues and identify treatment targets before the procedure, dynamically monitor the needle’s trajectory during the procedure to avoid damaging critical nerves, blood vessels, or tendons, and utilize ultrasound post-treatment to assess changes in imaging to evaluate therapeutic outcomes. Building on our team’s previous research foundation (Liu et al., 2022; Xiu et al., 2023). We hypothesized that ultrasound-guided acupotomy therapy, by loosening fibrotic lesions in the rectus femoris, could alter its pathological structure and slow the progression of KOA. We demonstrated that ultrasound-guided acupotomy therapy could reduce collagen volume fraction, decrease inflammatory cell infiltration in muscle fibrosis, increase average cross-sectional area, and restore the alignment of muscle fibrosis. Using ultrasound-guided acupotomy therapy for incising and needling the muscle tissue of the quadriceps at fibrotic lesion sites effectively released muscle tension, improved the muscle’s mechanical environment, and significantly alleviated inflammation in the rectus femoris of KOA rabbits, reducing muscle atrophy states (Liu et al., 2022; Oo et al., 2018). Other scholars have also confirmed that acupotomy can promote the repair of quadriceps fibrosis, enhance the contraction ability of the quadriceps, and adjust the biomechanical properties of knee tendons and ligaments (Ma et al., 2021). As cartilage degeneration is a characteristic pathological change in KOA, we further observed the morphology of KOA cartilage. Our findings confirmed that ultrasound-guided acupotomy therapy could reduce the degree of cartilage damage in the knee joints of KOA rabbits. To further support the specific molecular mechanisms by which acupotomy therapy can alter the pathological structure of the rectus femoris, we measured the extracellular matrix deposition markers α-SMA and VIM. As expected, acupotomy reduced their mRNA expression, therefores decreasing the degree of fibrosis in the rectus femoris of KOA. Therefore, our study confirms that visual acupotomy interventions, by loosening the fibrotic lesions in the rectus femoris, can effectively improve fibrosis in the rectus femoris and delay the progression of KOA. Studies have shown that the development of skeletal muscle fibrosis is closely linked to the TGF-β/Smad signaling pathway (Yao et al., 2024). This pathway facilitates the transition of myofibroblasts into fibroblasts and promotes the deposition of extracellular matrix components, such as collagen, thereby driving the onset and progression of fibrosis. Similarly, YAP (Yes-associated protein), as a key regulatory factor (Chu and Quan, 2024), enhances fibroblast activity during injury repair, and its dysregulation can lead to excessive fibrosis. Additionally, the PI3K/Akt signaling pathway regulates myocyte proliferation, differentiation, and survival, further promoting fibroblast activity and collagen deposition, which exacerbates fibrosis (Shamsan et al., 2024). These pathways may represent critical mechanisms by which acupotomy alleviates rectus femoris fibrosis and slows the progression of KOA cartilage degeneration, offering valuable directions for future mechanistic research.

We acknowledge several limitations in our study. First, as a preliminary short-term study, it involved only semi-quantitative analyses on a small sample size, focusing on the stiffness, cross-sectional area, and fibrosis of the rectus femoris. Further large-scale experimental and clinical studies are needed to better understand the chronic pathological changes of the rectus femoris. The study also lacked a stage-specific design for acupotomy interventions, as ultrasound-guided acupotomy therapy was not evaluated for different pathological stages of the rectus femoris in KOA. This limitation prevents us from determining the optimal timing and clinical protocols for the therapy. Moreover, the molecular mechanisms underlying the effects of acupotomy were not investigated, leaving an important aspect unexplored. Another challenge lies in the clinical translation of the findings. Extrapolating results from animal models to humans remains difficult, and many medical centers worldwide may lack the resources to implement this therapy effectively. Understanding the progression of chronic injury in the rectus femoris is crucial for formulating rehabilitation strategies for KOA and assessing disease prognosis. Future research directions worth exploring include the fibre types of the rectus femoris, multimodal ultrasound diagnostics, specific molecular mechanisms of visual acupuncture needle interventions, and optimization of clinical procedures.

## 5 Conclusion

In this study, we conducted a systematic investigation of the pathological characteristics of the rectus femoris in KOA rabbits at various stages, providing preliminary insights into the patterns of its pathological evolution throughout KOA progression. As the disease progresses, the elastic modulus of the rectus femoris increases, its cross-sectional area decreases, and the degree of fibrosis progressively intensifies. By using ultrasound-guided acupotomy therapy to mitigate the degree of fibrosis in the rectus femoris, a protective effect on the cartilage is achieved. These findings provide new insights into the repair of chronic injuries to the rectus femoris and offer novel rehabilitation treatment strategies for managing KOA.

## Data Availability

The original contributions presented in the study are included in the article/Supplementary Material, further inquiries can be directed to the corresponding author.
