# Supplementary material for: Characterization of rectus femoris lesions in knee osteoarthritis at different stages and the effect of ultrasound-guided acupotomy
Source: Front Physiol. 2025 Jan 23;15:1496425. doi: 10.3389/fphys.2024.1496425 (PMC11799896; doi:10.3389/fphys.2024.1496425)
Supplement: Supplementary file 1 [file Table1.docx]

**Supplementary Tables**

**Supplementary Table 1** Chemicals, reagents, and antibodies

| REAGENT or RESOURCE | SOURCE | IDENTIFIER |
| --- | --- | --- |
| HE and Masson's trichrome staining kit | Solarbio | G1340 |
| Glutaraldehyde | AlaAesar | F-A17876 |
| 4% paraformaldehyde | Solarbio | P1110 |
| 10% EDTA | Baiao Leibo | GL1794 |
| Automatic tissue dehydrator | Kanglong Electronic Technology | KL-T1 |
| Tissue embedding system | Zhongwei Electronic Instrument | BMJ-A |
| 3.0T high-field MRI scanner | Siemens AG |  |
| ultrasound diagnostic instrument | HI VISION Avius L |  |
| acupuncture needle | Laozongyi |  |
| Anti-α-SMA antibody | Boaosen | bs-10196R |
| Anti-VIM antibody (H5) | Boaosen | bs-8533R |

**Supplementary Table 2** RT-qPCR Primers used in this study.

| Name | Origin | Forward (5’→3’) | Reverse (5’→3’) |
| --- | --- | --- | --- |
| α-SMA | Rabbit | GTCAGGAATCCCGTGAAGCA | CATTGTCACACACAAGGGCG |
| VIM | Rabbit | TGGACATTGAGATCGCCACC | GAGTGGGTGTCAACCAGAGG |
| GAPDH | Rabbit | TGGAATCCACTGGCGTCTTCAC | AGGATGCGTTGCTGACAATCTTGA |

**Supplementary Table3** Acupotomy intervention and site selection

| Groups | | Intervention methods and Location of acupotomy |
| --- | --- | --- |
| Control | | Grabbing action only, no intervention |
| KOA 6weeks | | After successful modelling, only the grasping action is performed without intervention. |
| Acupotomy | After successful modelling, ultrasound-guided acupotomy treatment was performed once a week for a total of 4 weeks. ①“He Din Ci”(foot yangming meridian tendon focal point, attachment of quadriceps tendon to the positive upper edge of the patella)；②“Bin Wai” (foot yangming meridian tendon focal point，middle margin of lateral patella, lateral patellar support band, knee capsule case)；③“Bin Nei” (middle of the medial edge of patella, medial patellar support band, capsule of knee joint)；④“Bin Nei Xia” (the focal point of the foot yangming meridian tendon, the medial knee eye of the patellar ligament)；⑤“Bin Wai Xia”(foot yangming meridian tendon focal point, patellar ligament lateral external knee eye)；⑥“Yin Ling Ci” (the focal point of san yin meridian tendons of the foot, at the medial tibial stop of the goosefoot tendon). Three to five of these points were selected for visualized needle-knife release each time. | |
